# Human cytomegalovirus (CMV) dysregulates neurodevelopmental pathways in cerebral organoids

**DOI:** 10.1038/s42003-024-05923-1

**Published:** 2024-03-19

**Authors:** Ece Egilmezer, Stuart T. Hamilton, Charles S. P. Foster, Manfred Marschall, William D. Rawlinson

**Affiliations:** 1 Serology and Virology Division, Microbiology, NSW Health Pathology, Prince of Wales Hospital, Sydney, NSW 2031 Australia; 2 School of Medical Science, University of New South Wales, Sydney, NSW 2052 Australia; 3 School of Clinical Medicine, University of New South Wales, Sydney, NSW 2052 Australia; 4 Institute for Clinical and Molecular Virology, Friedrich-Alexander University of Erlangen-Nürnberg, Erlangen, 91054 Germany; 5 School of Biotechnology and Biomolecular Sciences, University of New South Wales, Sydney, NSW 2052 Australia

**Keywords:** Virology, Pathogenesis

## Abstract

Human cytomegalovirus (CMV) infection is the leading non-genetic aetiology of congenital malformation in developed countries, causing significant fetal neurological injury. This study investigated potential CMV pathogenetic mechanisms of fetal neural malformation using in vitro human cerebral organoids. Cerebral organoids were permissive to CMV replication, and infection dysregulated cellular pluripotency and differentiation pathways. Aberrant expression of dual-specificity tyrosine phosphorylation-regulated kinases (DYRK), sonic hedgehog (SHH), pluripotency, neurodegeneration, axon guidance, hippo signalling and dopaminergic synapse pathways were observed in CMV-infected organoids using immunofluorescence and RNA-sequencing. Infection with CMV resulted in dysregulation of 236 Autism Spectrum Disorder (ASD)-related genes (*p* = 1.57E-05) and pathways. This notable observation suggests potential links between congenital CMV infection and ASD. Using DisGeNET databases, 103 diseases related to neural malformation or mental disorders were enriched in CMV-infected organoids. Cytomegalovirus infection-related dysregulation of key cerebral cellular pathways potentially provides important, modifiable pathogenetic mechanisms for congenital CMV-induced neural malformation and ASD.

## Abstract (web summary)

Utilising the in vitro multicellular human cerebral organoid model and RNA sequencing analysis, cytomegalovirus (CMV) -induced dysregulations in neuronal gene systems and pathways were identified, including a possible association between congenital cerebral CMV infection and Autism Spectrum Disorder (ASD).

## Introduction

Human cytomegalovirus (CMV) infection is a major cause of congenital malformation in developed countries, with 10–15% of fetal CMV infections resulting in symptomatic congenital disease. Approximately 0.5% of these cases result in fetal death, and 10–15% of asymptomatic congenitally infected neonates develop significant clinical sequelae later during infancy^1,2^. The clinical outcomes of congenital CMV infection of the fetal brain include microcephaly, intracranial calcifications, cerebral palsy, mental disability, sensorineural hearing loss, seizures, and visual impairment^3–5^. The pathogenetic mechanisms of CMV-induced central nervous system damage have not yet been fully elucidated.

In this study, we utilised multicellular cerebral organoids to demonstrate CMV-induced changes and interrogated the accompanying mRNA and protein dysregulation. Cerebral organoids are an in vitro, three-dimensional and multi-cell type model of the human brain. They mimic in vivo developmental events and have the capability of self-organising into different cerebral regions. These organoids have been used to model neurodevelopmental phenotypes such as microcephaly, and disease modelling in CMV and Zika virus infection^6–10^. Infection of cerebral organoids with CMV has been previously shown to induce neuropathological changes similar to that seen with clinical infection. The observed pathology of infected organoids included abnormal calcium signalling, aberrant cortical layer formation, and dysregulated expression of the neural marker β-III tubulin^9,11^.

Dysregulation of key cellular neurodevelopmental signalling pathways is a potential outcome of CMV infection causing fetal neural injury. We have previously shown dual-specificity tyrosine phosphorylation-regulated kinases (DYRKs) play a critical role in CMV replication in fibroblast, placental trophoblast and placental ex vivo explant cultures^12,13^. The DYRK family are key regulators of cell growth and differentiation, and modulate signalling pathways including sonic hedgehog (SHH)^14,15^. We have also shown CMV-infection of human foreskin fibroblast cells (HFF) up-regulates expression of SHH family proteins including ULK3, Gli2 and Rb^12^. The SHH pathway proteins function to regulate neural progenitor cell proliferation, neural progenitor differentiation, midbrain and neural tube pattern formation, synapse formation, assisting to promote blood-brain barrier integrity^16–20^.

Using infection of human cerebral organoids to assess protein changes, we show CMV infection dysregulates localisation and expression of DYRK pathway (DYRK1A, DYRK1B) and SHH pathway (Shh morphogen, Gli2 transactivator, ULK3 kinase, and Rb tumor suppressor protein) proteins. Using RNA-seq analysis of differentially expressed genes (DEG), we show CMV infection may induce aberrant neural differentiation and dysregulation of key neurodevelopmental pathways, particularly those involved in neurogenesis and cellular differentiation. There was a moderate association between DEGs of CMV infected cerebral organoids and genes linked to the development of Autism spectrum disorder (ASD). These data generate a testable hypothesis for future studies investigating the potential causative nature of CMV in ASD.

## Results

### Organoids generated from human induced pluripotent stem cells (iPSCs) displayed multiple markers for cerebral tissue and supported CMV infection and replication

**Fig. 1:**
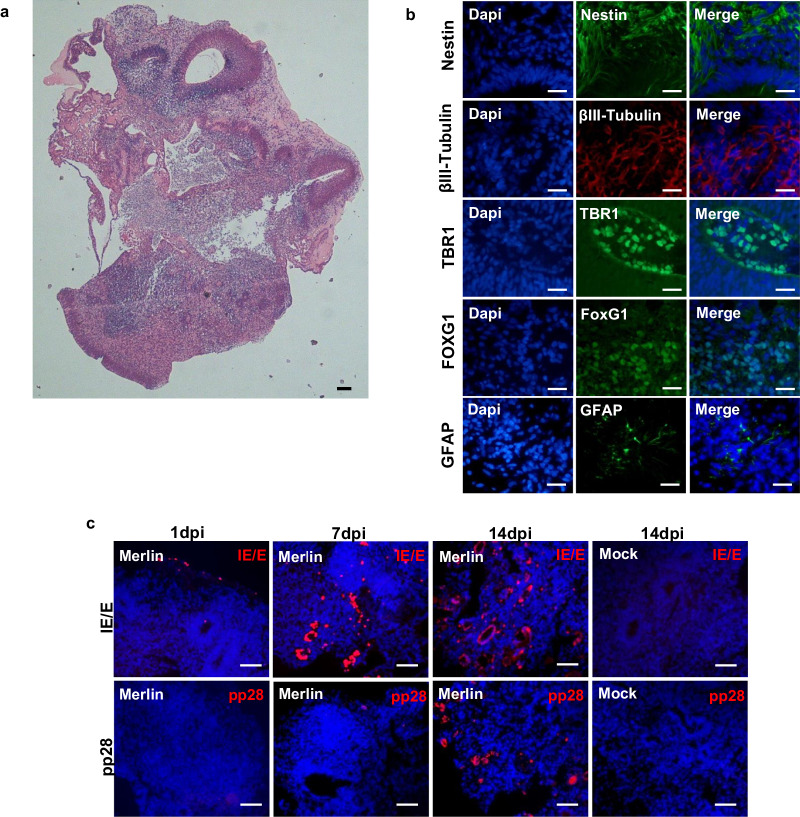
Organoids generated from human induced pluripotent stem cells (iPSCs) displayed multiple markers for cerebral tissue and supported CMV infection and replication. **a** H&E staining of Mock-infected cerebral organoids at 1dpi. **b** Immunofluorescence staining of FFPE organoid sections at 1dpi; Nestin (neural progenitor cells), βIII-Tubulin (neurons), TBR1 (deep-layer neurons and cerebral pre-plate), FOXG1 (forebrain), and GFAP (astrocytes). Scale bars represent 25 µm. **c** Immunofluorescence of FFPE cerebral organoids infected for 1, 7, and 14 days. Staining for CMV immediate early/early protein (IE/E) shows susceptibility of cerebral organoids to CMV infection and spread of infection with increased IE/E expression from day 1–14. Staining for the late CMV protein (pp28) shows the ability of cerebral organoids to support viral replication. Scale bars represent 100 µm.

Staining with H&E of cerebral organoids generated from iPSCs^21^ revealed a complex structural diversity at 1dpi following 55 days of culture. The visible structures resembled neuroepithelial rosettes and tubules (Fig. 1a). The organoids also expressed Nestin, a marker of neural progenitor cells and βIII-Tubulin, an indicator of neuronal differentiation. The presence of TBR1 positive cells indicated development of deep-layer neurons. At 1dpi following 55 days of cerebral organoid culture, small regions of GFAP positive cells were observed, a marker of astrocytes. At this stage of culture, different brain regions had developed with Foxg1-positive staining marking forebrain development (observed from the periphery to the centre of a circular region of the organoid) and TBR1-positive staining indicating the development of the pre-plate, pre-cursor to the cerebral cortical plate (Fig. 1b).

Cerebral organoid sections were stained for CMV IE/E (immediate early/early) and CMV pp28 (true late) proteins at 1, 7, and 14 days post infection (dpi) (Fig. 1c). At 1 dpi, CMV-infected organoids showed a small number of IE/E positive cells (~0.05–0.1% infection), indicating the cerebral organoids were susceptible to CMV infection. By 7dpi, CMV-infected organoids showed dissemination of IE/E positive cells (~5–10% infection) with cells also expressing pp28 true late protein, indicating cerebral organoids support the full CMV replication cycle. At 14dpi, there was clear dissemination of CMV throughout the cerebral organoids with a noticeable increase in IE/E (~30% infection) and pp28 positive cells. Furthermore, by 14dpi, the IE/E positive cells were observed to be spread in clusters throughout the organoid, indicating the cells were infected by neighbouring cells. There was no staining of CMV IE/E or pp28 proteins observed in mock-infected organoids.

To determine the cell populations that were permissive to CMV infection in the cerebral organoids, immunofluorescence was performed on 14 day post infection Merlin-infected FFPE organoid sections, co-staining IE/E with Nestin, GFAP, or VGluT1 (Supplementary Fig. 1). Some regions of Nestin and VGluT1 positive cells were also IE/E positive, suggesting CMV infected neural progenitor cells and excitatory neurons respectively. Very limited regions of GFAP positive cells co-stained with IE/E, indicating astrocytes were not preferentially infected with CMV by 14 days post infection.

### RNA-seq reveals CMV infection of cerebral organoids dysregulates cellular pluripotency and differentiation protein pathways

**Fig. 2:**
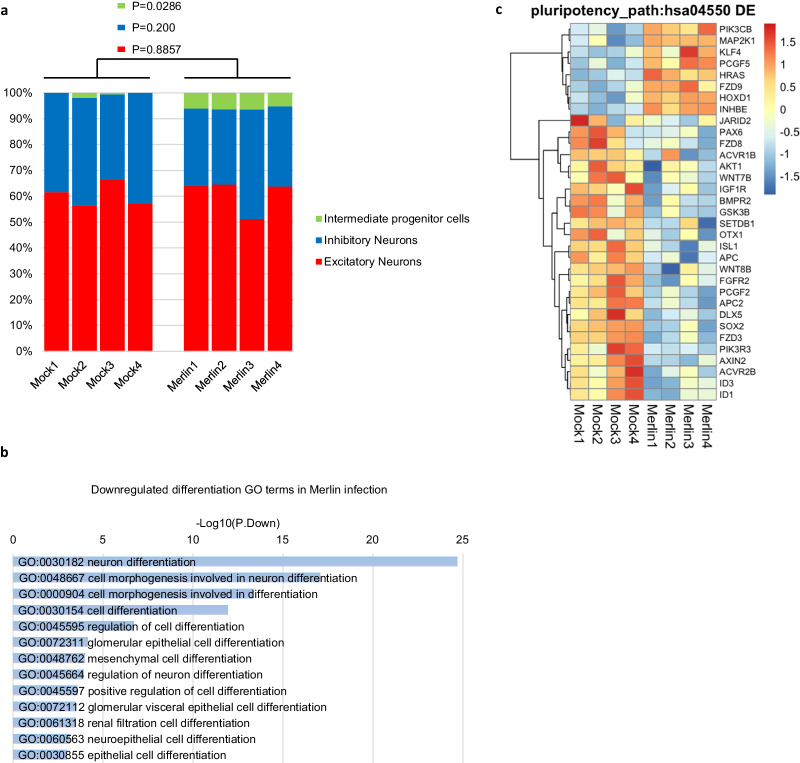
RNA-seq reveals CMV infection of cerebral organoids dysregulates cellular pluripotency and differentiation. **a** De-convolution analysis revealing the different cellular populations in generated cerebral organoids. **b** Downregulated DEGs with an FDR cut-off in CMV-infected cerebral organoids relative to mock were over-represented in the GO terms associated with cellular differentiation. **c** Heatmap of KEGG signalling pathways regulating pluripotency of stem cells (hsa04550) displaying genes upregulated and downregulated in CMV-infected compared to mock-infected organoids. See also Supplementary Data 1, Supplementary Data 2, and Supplementary Data 3.

We analysed a previously published single cell RNA-seq dataset of cerebral organoids to ensure our samples in the present study possessed a gene expression signature typical of cerebral organoids^22^. This analysis revealed that organoid samples generated for our study consisted primarily of Excitatory Neurons, Inhibitory Neurons, and Intermediate Progenitor Cells, with similar percentages of these cells in each experimental sample. On average, the group of CMV-infected organoids exhibited a 14.8% reduction in inhibitory neurons compared to the group of mock-infected organoids (*P* = 0.200). There was no significant difference between the groups for excitatory neuron populations (0.6% increase in the group of CMV-infected organoids compared to mock-infected organoids; *P* = 0.8857). There was a strong retention of intermediate progenitor cells in the CMV-infected organoid group with 4/4 of the CMV-infected organoids retaining intermediate progenitor populations (mean of 6.02% ±  0.49% within the group), whereas only 2/4 of the mock-infected organoids still retained intermediate progenitor cells (mean of 1.27% ± 0.72%) (*P* = 0.0286) (Fig. 2a).

Differential expression analysis revealed that of the 4029 differentially expressed genes (DEGs), 2208 were upregulated in CMV-infected organoids relative to non-infected organoids, and 1821 downregulated (Supplementary Data 1). All enrichment results including GO and KEGG analyses are based on the analyses without a fold-change cutoff. We tested whether the DEGs were significantly enriched for gene ontology (GO) terms or KEGG pathways. The major, and the more subtle CMV-induced transcriptional changes were assessed without implementing a fold-change threshold, using statistical significance based on FDR values only. This was to identify trends, and to account for the relatively low number of infected cells at 14dpi (Fig. 1c), a factor that would decrease the potential detection of statistically significant change in DEGs.

The GO analysis revealed over-representation of downregulated DEGs in CMV-infected organoids relative to mock that are known to be associated with cellular differentiation (*P* < 0.05) (Fig. 2b, Supplementary Data 2). These terms included neuron differentiation, cell morphogenesis involved in neuron differentiation, regulation of neuron differentiation, cell morphogenesis involved in neuron differentiation, regulation of cell differentiation, and cell differentiation. KEGG pathway analysis of significantly downregulated DEGs in CMV-infected organoids relative to mock revealed significant over-representation of KEGG signalling pathways regulating pluripotency of stem cells (hsa04550) (Fig. 2c, Supplementary Data 1).

### Key cellular genes and pathways are dysregulated in CMV-infected cerebral organoids

**Fig. 3:**
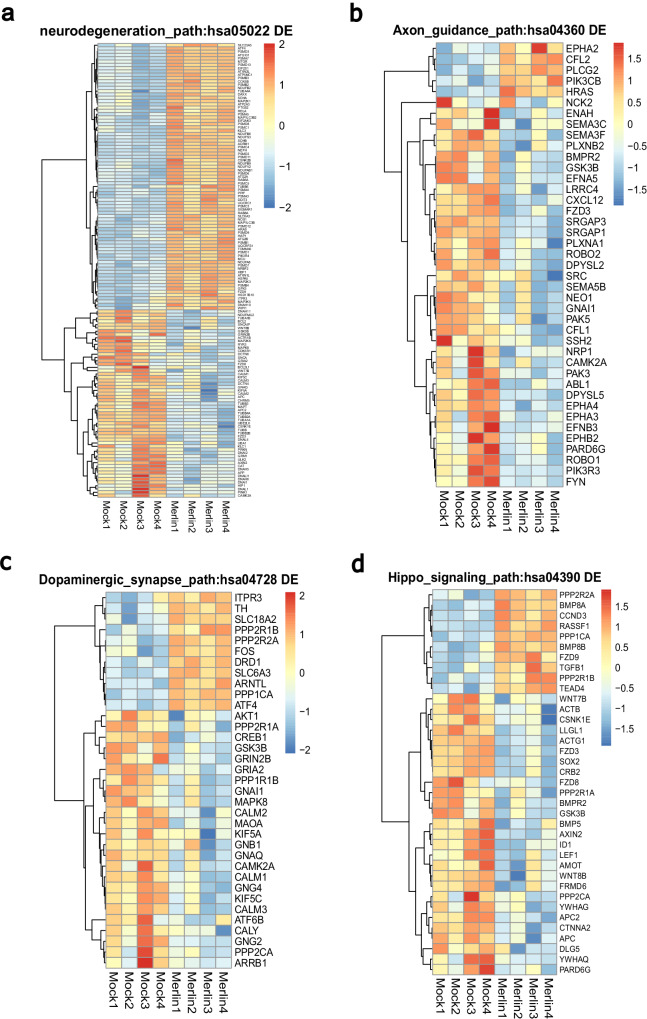
Key cellular pathways are dysregulated in CMV-infected cerebral organoids. Heat maps of key KEGG pathways involved in cerebral development in DEGs of CMV-infected organoids with FDR cut-off. **a** Pathways of neurodegeneration - multiple diseases (hsa05022). **b** Axon guidance pathway (hsa04360). **c** Dopaminergic synapse pathway (hsa04728). **d** Hippo signalling pathway (hsa04390). See also Supplementary Fig. 1, Supplementary Data 1, and Supplementary Data 3.

Upregulated DEGs in CMV-infected organoids relative to mock were over-represented in several critical KEGG pathways including pathways of neurodegeneration-multiple diseases (hsa05022) (Fig. 3a). Downregulated DEGs were over-represented in KEGG pathways, axon guidance (hsa04360) (Fig. 3b), dopaminergic synapse (hsa04728) (Fig. 3c), and hippo signalling (hsa04390) (Fig. 3d) (Supplementary Data 3). The KEGG pathways in genes that were upregulated in CMV-infected organoids relative to mock Alzheimer disease (hsa05010), Parkinson disease (hsa05012), and Huntington disease (hsa05016), were all significantly over-represented (Supplementary Fig. 1, Supplementary Data 3).

**Fig. 4:**
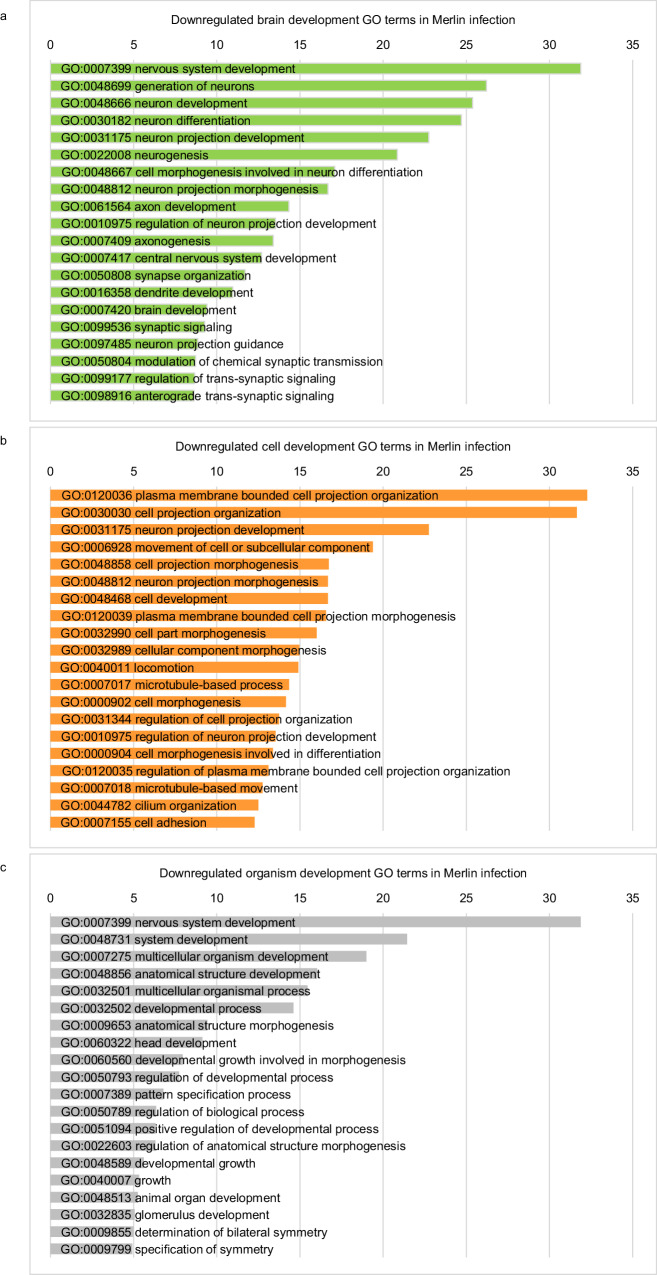
Key cellular genes are dysregulated in CMV-infected cerebral organoids relative to mock. Analysis of downregulated DEGs that are over-represented in GO systems. **a** Top 20 GO terms of downregulated DEGs involved in brain development. **b** Top 20 GO terms of downregulated DEGs involved in cell development. **c** Top 20 GO terms of downregulated DEGs involved in organism development. See also Supplementary Data 2.

Downregulated DEGs in CMV-infected organoids compared to mock were over-represented in GO terms associated with brain development including GO:0030182-neuron differentiation, GO:0048666-neuron development, and GO:0048699-generation of neurons (Fig. 4a), cell development including GO:0120036-plasma membrane bounded cell projection organization, GO:0030030-cell projection organization, and GO:0048468-cell development (Fig. 4b), and organism development including GO:0007399-nervous system development, GO:0048731-system development, and GO:0007275-multicellular organism development (Fig. 4c).

### CMV infection of cerebral organoids results in re-localisation of DYRK and SHH proteins but no strong change in gene expression

**Fig. 5:**
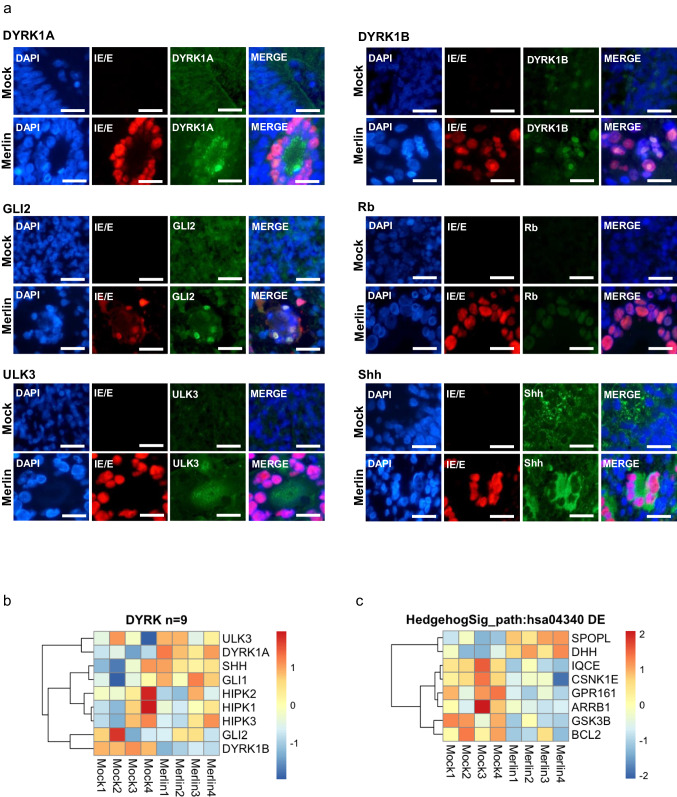
CMV infection of cerebral organoids results in re-localisation of DYRK and SHH proteins but no strong change in gene expression. Cerebral organoids at 55 days of age were infected for 14 days with CMV (Merlin strain). **a** Immunohistochemistry was performed on FFPE sections. CMV infection induced re-localisation of DYRK and SHH proteins in cerebral organoids, particularly in regions of syncytia. CMV induced re-localisation of DYRK1A, ULK3, and Shh to the cytoplasm and DYRK1B, GLI2, and Rb to the nucleus. Scale bars represent 25 µm. **b** Organoids were infected with CMV or left untreated (mock) in quadruplicate for bulk RNA-sequencing. Heat map of the 9 DYRK-associated genes that were identified in CMV-infected organoids through RNA-sequencing. **c** Heat map of the 8 significantly differentially expressed SHH genes. See also Supplementary Data 1, Supplementary Data 4.

Expression of DYRK and SHH proteins was investigated in CMV-infected cerebral organoids relative to mock uninfected organoids. In uninfected organoids, each of the proteins were diffusely expressed with DYRK1A, DYRK1B, Gli2 and ULK3 expression observed in both the nucleus and cytoplasm, Rb expression predominantly in the nucleus, and Shh observed in the cytoplasm with some punctate expression throughout. In CMV-infected organoids, particularly in areas of CMV-induced syncytia, there was clear re-localisation of the cellular proteins in the centre of the cytoplasmic syncytial region. DYRK1A, ULK3, and Shh were re-localised to the cytoplasm. DYRK1B, GLI2, and Rb re-localised to the nucleus of infected cells with increased expression observed only within the infected cells (Fig. 5a).

We were particularly interested in understanding the effects of CMV-infection on DYRK and SHH gene transcription, as we have shown these altered previously in other studies^12,13^. The expression of DYRK and HIPK genes, both from the DYRK family of proteins, were investigated in mock- and CMV-infected organoids. Of the 9 genes from the DYRK family, only DYRK1B exhibited a modest downregulation in CMV-infected organoids (Fig. 5b, Supplementary Data 4).

Although the Sonic Hedgehog KEGG pathway was not found to be significant in our KEGG pathway analysis, we note that 8 genes in this pathway were differentially expressed (6 downregulated, 2 upregulated) (Fig. 5c, Supplementary Data 4). Of note, the SHH effector RB1 was found to be significantly upregulated in CMV infected organoids relative to mock (Supplementary Data 1).

### Differentially expressed genes (DEGs) in CMV-infected cerebral organoids were found moderately associated with autism spectrum disorder (ASD)-related genes and associated with other neurological and congenital disorders

**Fig. 6:**
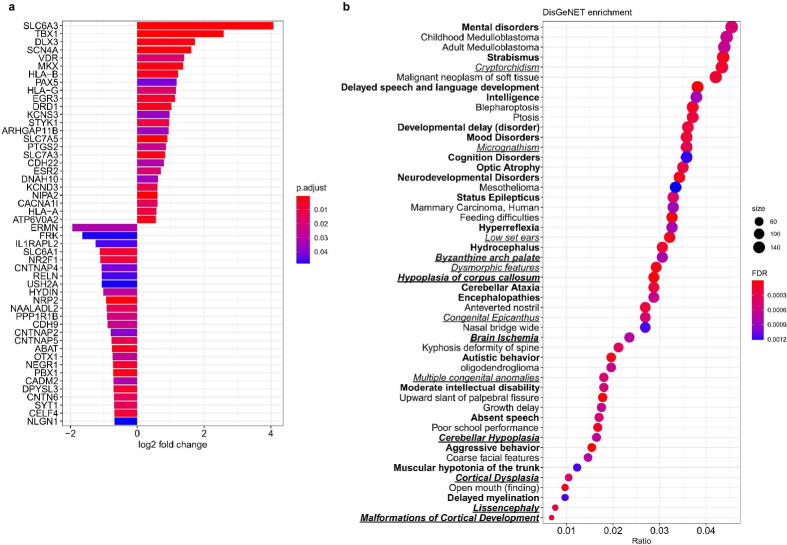
Differentially expressed genes in CMV-infected cerebral organoids are moderately associated with autism spectrum disorder (ASD)-related genes and are implicated in several neurological and congenital diseases and symptoms. **a** A hypergeometric test was performed on differentially expressed genes to identify genes overlapping with the SFARI database. A total of 236 genes were found to be significantly (*p* = 1.57E-05) upregulated or downregulated in cerebral organoids infected with CMV. The top 25 most upregulated and downregulated (log2 fold change) is shown in this graph. See also Supplementary Data 5. **b** Disease enrichment of differentially expressed genes between CMV-infected and mock-infected organoids was tested using the DisGeNET database. The top 50 enriched diseases and symptoms that were driven by at least 25 genes were plotted based on FDR against the “ratio” (count of DE genes assigned to a disease)/(total number of DE genes). Nervous system disease, mental disorders, or behavioural symptoms are in bold and congenital abnormalities are italicised and underlined. See also Supplementary Data 6.

The representation of DEGs from the bulk sequencing data set generated from mock- and CMV-infected cerebral organoids were compared with the autism spectrum disorder (ASD) Simons Foundation Autism Research Initiative (SFARI) database using a hypergeometric overlap test. We found a significant over-representation of ASD-related genes in our list of DEGs (236 overlapping DEGs, *p* = 1.57E-05) (Fig. 6a, Supplementary Data 5). The specificity of this association was assessed using an additional hypergeometric overlap test to compare with datasets of genes from conditions not known (and unlikely to be on first principles) to be associated with CMV infection. There was no significant over-representation or under-representation of Schizophrenia-associated genes when compared with the DEGs altered in CMV infection of cerebral organoids.

Further assessment of the specificity of association between CMV infection associated DEGs and other conditions was performed by testing for disease enrichment in our DEGs dataset using the DisGeNET database. This identified that our DEGs were significantly enriched in 240 diseases and syndromes (FDR < 0.05). Of these, 103/240 were related to nervous system disease, mental disorders, or behavioural symptoms, including “Hypoplasia of corpus callosum” (FDR = 7.87E-07), “Delayed speech and language development” (FDR = 7.87E-07), “Developmental delay (disorder)” (FDR = 2.01E-04), “Autistic behaviour” (FDR = 7.09E-05), “Neurodevelopmental Disorders” (FDR = 7.09E-05), “Status Epilepticus” (FDR = 4.12E-04), and “Progressive microcephaly” (FDR = 2.94E-02). Congenital abnormalities accounted for 52/240 of the significantly enriched diseases (Fig. 6b, Supplementary Data 6). This suggests some overlap in gene expression following CMV infection of cerebral organoids, and conditions with similar clinical phenotypes such as cerebral hypoplasia, neurodevelopmental delay, seizures and microcephaly^23^.

## Discussion

Human CMV infection of the fetal brain can result in the development of serious neurological sequelae, although lack of knowledge around mechanisms of neural malformation reduce the potential for therapeutic interventions. In this study, using the Merlin strain of human CMV (genetically intact), we show (i) cerebral organoids were permissive to full cycles of CMV replication, (ii) CMV infection dysregulated neuronal cellular pluripotency and differentiation in organoid models, (iii) CMV infection of cerebral organoids resulted in dysregulation of several neurodevelopmental systems including neurodegeneration, axon guidance, and synapse pathways, (iv) CMV induced alteration of DYRK and SHH protein localisation without clear change in mRNA levels, and (v) moderate association of CMV infected cerebral organoids with ASD syndrome genes, particularly genes known to be linked with neuronal development.

Two-dimensional cell monocultures used in assessment of infection effects do not take into account the specific gene regulation patterns of different cell populations within the human brain^24–26^. Animal models, although consisting of multiple cell and organ types, are not able to accurately represent human CMV infection due to the species-specific nature of the virus^27^. Congruent with previous studies of Lancaster et al., the organoids generated here developed complex structures, different cerebral regions, and different cell types, displaying the three-dimensional organisation which resembled in vivo brain development.

Bulk RNA-seq analysis of cerebral organoids suggested CMV infection dysregulated genes and pathways that are critical for fetal brain development. The data indicate one potential mechanism for fetal neuronal injury is through dysregulation of functions involving preservation of neuronal integrity. Neurodegenerative diseases, including Alzheimer’s, Parkinson’s, and Huntington’s disease, involve the loss of neurons as well as changes in their pattern and distribution^28,29^. These three neurodegenerative diseases and pathways of neurodegeneration were all significantly over-represented in upregulated DEGs of CMV-infected cerebral organoids. Although these disease often present later in life, over-representation of these disease pathways, suggests a reduction in neuronal populations in the fetal brain from CMV infection. Consistent with this, de-convolution analysis revealed all four CMV-infected organoids retained a population of intermediate progenitor cells. However, only two of the four mock-infected organoids retained a minimal population of intermediate progenitors, albeit a non-significant difference, which may be due to limited sample size (*n* = 4). Intermediate progenitor cells are located in the subventricular zone of the brain and differentiate into daughter neurons^30^. A possible mechanism for the CMV-induced retention of this population of cells is through disrupted neuronal differentiation. Gene ontology analysis corroborates this, given our data show significant CMV-induced over-representation of downregulated DEGs in systems involved in cellular differentiation. These data suggest CMV infection may acutely induce neurodegenerative disease-like pathology through a decrease in neuronal cell population by attenuating neuronal differentiation in the developing fetal brain.

The significant alteration of protein localisation for DYRK (DYRK1A, DYRK1B) and SHH (ULK3, GLI2, Shh, Rb [effector]) in CMV-infected cerebral organoids relative to mock is consistent with previous observations in HFF cells^12^. Dysregulation of DYRKs have been reported in various neurodevelopmental phenotypes where haploinsufficiency of DYRK1A has been linked to the development of microcephaly, intellectual disabilities, and developmental delay, similar to clinical manifestations of congenital CMV^3–5,31–33^. Similarly, the SHH genes GLI2 and Shh gene are associated with Holoprosencephaly-like phenotypes which are characterised by a lack of midline division of the forebrain^34,35^. Furthermore, recent studies have implicated DYRK1A and the SHH pathway as risk factors in Autism spectrum disorder (ASD)^36–38^. Therefore, CMV-induced dysregulation of DYRK and SHH protein expression may be a mechanism for the development of cerebral malformations, providing a mechanistic link between congenital infection and the development of cerebral clinical sequelae. However, despite the observed significant CMV-induced DYRK and SHH protein changes, this study only found a statistically significant decrease in DYRK1B expression and increase in RB1 expression in CMV infected organoids relative to mock. It is possible that the observed changes in protein localisation may be due to protein accumulation as opposed to transcriptional changes.

Our data suggest CMV induces cerebral malformation by dysregulating genes associated with neurodevelopment including nervous system development, head development, and brain development GO terms, corroborating some of the findings in a study reporting RNA sequencing analysis in CMV-infected (TB40/E expressing EGFP strain) cerebral organoids, sorting GFP+ and GFP- populations of cells^10^ Interestingly, O’Brien et al. also reported GFP (Low) cells exhibited similar transcriptional effects as GFP (+) populations, suggesting CMV infection affects cells despite no evidence of active viral replication^10^. Furthermore, we reported 103 of the 240 diseases and syndromes derived from the DisGeNET database that were significantly enriched in our list of DEGs were related to nervous system disease, mental disorders, or behavioural symptoms, including “autistic behaviour” (FDR = 7.09E-05). Autism spectrum disorder (ASD) is a neurodevelopmental condition that is described by deficits in communication, restricted interests, and behavioural issues^39^. The increase in ASD diagnoses worldwide has highlighted the importance of investigating factors that cause this condition and associated comorbidities including epilepsy, learning and intellectual disabilities, and sensory problems^40,41^.

In support of the ASD association suggested by our DisGeNET disease enrichment analyses, our hypergeometric analyses using the SFARI database also found an overlap of 236 ASD-related genes with those genes found to be differentially expressed in this study, representing a significant over-representation. These findings are the first evidence suggesting a moderate and possible association between congenital CMV infection and the development of ASD or ASD-like phenotypes. To further corroborate the theory that CMV infection may be associated with ASD-like phenotypes, a number of behavioural symptoms associated with ASD (as outlined in the DisGeNET database) were significantly enriched in our list of differentially expressed genes, including “Delayed speech and language development”, “Moderate intellectual disability”, and “Severe intellectual disability”.

Neuronal circuits in the brain are composed of a highly-regulated ratio of excitatory and inhibitory neurons^42^. This study shows a reduced proportion of inhibitory neurons in CMV-infected organoids relative to mock uninfected organoids, using de-convolution analysis. Interestingly, epilepsy which is an ASD comorbidity, has recently been associated with congenital CMV^40,43^. The DisGeNET diseases “Seizures, Focal”, “Visual seizure”, “Myoclonic Seizures”, “Complex partial seizures”, “Status Epilepticus”, and “Epilepsy, Rolandic” were all significantly enriched in the list of genes differentially expressed between CMV-infected and mock-uninfected organoids. A mechanism of epilepsy is the abnormal firing of excitatory neurons caused by the absence of GABAergic inhibition in specific brain regions^44–46^. Reduced proportions of GABAergic (inhibitory) interneuron subsets and an increase in seizure susceptibility were reported in an ASD mouse model^47^. The decrease in inhibitory neurons observed in CMV-infected organoids is therefore an interesting potential mechanistic link between CMV and seizures observed as a clinical sequela of congenital CMV infection.

Our study indicates CMV-induced dysregulation of genes significantly enriched for GO terms including synapse organization, regulation of synapse structure or activity, and synapse assembly, as well as the dopaminergic synapse KEGG pathway. The axon guidance pathway, which is responsible for facilitating movement of axons to their target locations to form synaptic connections^48^, was over-represented in downregulated DEGs in CMV infected organoids relative to mock uninfected organoids. Mouse models have similarly shown CMV infection of neurons impaired synaptic activity^49,50^. Synaptic homeostasis is critical for typical neuronal connectivity, and it has been hypothesised that dysregulation in this homeostasis increases the risk for ASD. Mutations in ASD-risk genes impair synaptic function and their prevalence which results in atypical neuronal connectivity^51–53^. The CMV-induced dysregulation of genes enriched for GO terms and KEGG pathways associated with synaptic function suggests a possible mechanism for neuronal injury, similar to those observed in ASD-like phenotypes.

This study provides evidence that suggests CMV dysregulates significant neurodevelopmental pathways. Utilising the in vitro multicellular human cerebral organoid model and RNA sequencing analysis, novel CMV-induced dysregulations in neuronal gene systems and pathways were determined, including a possible association between congenital cerebral CMV infection and ASD. Furthermore, by identifying putative disease associations with CMV infection utilising the DisGeNET database, we were able to generate testable hypotheses for future studies. This model can be used in future studies to investigate novel and commercially available therapeutics and their effects on resolving CMV-induced dysregulations of cerebral gene and protein expression.

## Methods

### Cell lines and preparation of virus stocks

Human episomal induced pluripotent stem cells with a normal karyotype (Human Episomal iPSC line; Gibco) were cultured on vitronectin (VTN-N; Gibco) coated 6-well plates in complete Essential 8 Medium (Gibco) as per the manufacturer’s protocol. The medium was supplemented with 1X Revitacell (Gibco) for the first 24 h of passage. For the continued maintenance of the iPSCs, WISC Bank protocols for the culture of feeder independent iPSCs were used^21^. Briefly, the cells were passaged on Growth Factor Reduced Basement Membrane Matrix Matrigel (Corning) coated plates in complete mTeSR1 Medium (Stem Cell Technologies) and supplemented with ROCK Inhibitor (Y-27632Sigma-Aldrich) for the first 24 h of culture. Cell lines were Mycoplasma free and maintained at 37 °C with 5% CO2. The CMV strain Merlin (UL128+, RL132−) was propagated as previously described^21^. Viral titres were determined using standard plaque assays.

### Generation of cerebral organoids from iPSCs

Cerebral organoids were generated from iPSC, as described in detail by Lancaster et al.^21^. Briefly, embryoid bodies were generated by seeding 9,000 live iPSCs in low-bFGF hESC medium in a low-attachment 96-well U-bottom plate. The embryoid bodies were cultured for 5–7 days, with half the medium removed and replaced with fresh media every other day. ROCK inhibitor and bFGF were only included for the first 4 days. Primitive neuroepithelial cell clusters were generated by transferring the embryoid bodies to a low-attachment 24-well plate and cultured for 4–5 days in neural induction medium. Every 48 h of culture, fresh neural induction medium was added to each well. After 4–5 days of culture, the neuroepithelial tissues were embedded in Matrigel droplets and transferred to Petri dishes containing cerebral organoid differentiation medium without vitamin A. Following 48 h of culture, the medium was replaced with fresh cerebral organoid differentiation medium without vitamin A. After 4 days in culture, the Matrigel-embedded organoids were transferred to Petri dishes containing cerebral organoid differentiation medium containing vitamin A. The Petri dishes were transferred to an orbital shaker and the medium was replaced every 3–4 days with cerebral organoid differentiation medium containing vitamin A. The organoids were cultured for 55 days before undergoing mock or CMV infection and confirmation of cerebral differentiation of the organoid.

### Cerebral organoid infection with CMV

Cerebral organoids were inoculated 55 days after the induction of embryoid body growth with 1 × 10^7^ pfu of CMV Merlin and incubated at 37 °C with 5% CO_2_ on an orbital shaker, yielding an MOI of ~0.1, consistent with our previous work with CMV-infected placental explants^13^. After 24 h, the organoids were transferred to new dishes and the medium was replaced with fresh cerebral organoid media supplemented with vitamin A. The medium was changed every 3–4 days and the organoids harvested at days 1, 7, and 14 post-infection.

### Immunohistochemistry

Formalin fixed paraffin embedded (FFPE) cerebral organoid sections of 4 µm were de-paraffinised and rehydrated followed by antigen retrieval using Tris-EDTA buffer pH 9.0 for 20 min at 95 °C. These sections were incubated for 1 h with primary antibodies for CMV detection with mouse mAb anti-HCMV immediate early (IE1p72) and early (pUL44) antibody cocktail (IE/E; clones DDG9 and CCH2; Dako), mouse mAb-pp28 (Abcam); cerebral markers with rabbit mAb-Nestin (Abcam), mouse mAb-beta III Tubulin (Abcam), rabbit pAb-GFAP (Abcam), rabbit pAb-FOXG1 (Abcam), rabbit pAb-TBR1 (Abcam), rabbit mAb-VGluT1 (Abcam); and DYRK and Sonic Hedgehog (SHH) proteins with rabbit pAb-DYRK1A (Abcam), rabbit mAb-DYRK1B (Abcam), rabbit mAb-Sonic Hedgehog (Abcam), rabbit mAb-ULK3 (Abcam), rabbit mAb-Rb (Abcam), rabbit pAb-Gli2 (Abcam). An Fc blocking step was not utilised in our methods as we have previously shown no differences in staining patterns observed with and without Fc blocking^13,54^. Sections were incubated with secondary antibodies Alexa Fluor 488 goat anti-mouse and 594 goat donkey anti-rabbit (Invitrogen; 1:1000 dilution) for 30 min. DAPI (Invitrogen) was added to each slide, mounted with coverslips and imaged as previously described^13^.

### RNA extraction

Cerebral organoids cultured for 55 days followed by 14 days infection with CMV Merlin were harvested for bulk RNA-sequencing. Four organoids per condition (Mock and Merlin-infected) were harvested separately for Bulk RNA-sequencing. Prior to RNA extraction, the organoids were extracted from the Matrigel matrix in which they were embedded by transferring each organoid into one well of a 48 well plate. Pre-chilled Corning Cell Recovery solution (Corning Cell Recovery Solution; in Vitro Technologies) was added to each well until the organoids were just covered in solution. With a cut 1 ml pipette tip, the organoids were pipetted up and down and then incubated at +4 °C for 20 min. The organoids were then centrifuged at +4 °C at 200x g for 30 s and supernatant was removed followed by 2 washes with ice-cold PBS. The RNA extraction was performed using the RNeasy mini kit (RNeasy Mini Kit; Qiagen) as per manufacturer’s instructions. The RNA extracts were quantified using a nanodrop and processed for total RNA-sequencing of human samples using the Illumina Stranded Total RNA with RiboZero Plus kit and a NextSeq 500 HO 2x75bp flowcell.

### RNA-seq analysis, statistics, and reproducibility

Four replicates of each sample (i.e. four Mock- and four Merlin-infected organoids) were performed for RNA-bulk sequencing and no data were excluded. The RNA-seq libraries were sequenced using an Illumina NextSeq 500 to produce 75 nt paired-end reads for each sample. Read integrity and quality was confirmed using FastQC (v0.11.8). The reads were then mapped to the Ensembl *Homo sapiens* genome (GRCh38). Mapping was performed with Subread (v 1.6.3)^55^. The featureCounts function of Subread was used to generate counts of reads uniquely mapped to annotated genes using the GRCh38.108 gtf file.

In order to verify that each of the organoid samples had similar cellular composition and could be compared without this being a biasing factor, we used previously published single cell RNA-seq data^22^ to create a gene expression signature for the cell types likely to be found in cerebral organoids. We used 10,202 cells from 65-day cerebral organoids (ERS3646335, ERS3646335) to create a gene signature for Excitatory Neurons, Inhibitory Neurons, Intermediate Progenitor Cells, Radial Glia, and “Glycolysis” cells. The genes were filtered to require a cpm > 1 in at least 20% of the cells belonging to a particular cell type, leaving 5428 genes. The cibersort R package was used to predict the proportion of these cell types in the organoids^56^.

Differential expression analysis was performed using edgeR (v 3.38.4)^57^. Lowly expressed genes (cpm < 0.5 in at least four samples) were filtered out, leaving 20677 Ensembl Ids (17769 genes with a unique HGNC symbol) for analysis. Differential expression was performed using the glmQLFit and glmQLFTest functions of edgeR. Differential expression analyses were performed without an explicit fold-change cutoff incorporated into the model. The Kyoto Encyclopedia of Genes and Genomes (KEGG) pathway and Gene Ontology enrichment analyses of differentially expressed genes were performed using the kegga and goana functions of limma, respectively (3.52.3)^58^. In all cases, differentially expressed genes (DEGs) were defined as those genes with a Benjamini-Hochberg corrected *p*-value (FDR) <0.05.

We tested whether there was a significant under- or overrepresentation of ASD-related genes in our list of DEGs using a hypergeometric test, implemented using the phyper function from the base stats package of R. For this analysis we used a curated set of ASD-associated genes that were downloaded from the SFARI-gene database (https://gene.sfari.org/database/gene-scoring/) and then filtered to retain only those genes that were detected via RNAseq in our study (*n* = 947). ASD-related differentially expressed genes were then plotted using the barplot function of the enrichplot R package^59^, visualising associated log(2) fold changes and adjusted *p*-values from the differential expression analysis.

As a form of comparison to assess specificity of the findings, we repeated the same analysis, but with the input comprising schizophrenia-associated genes derived from GWAS analyses^60^.

The hypergeometric tests that we conducted represent conservative tests of disease-associated enrichment. Accordingly, we also conducted a less conservative analysis by testing for disease enrichment in the study DEGs using the disgenet2r R package, which queries the DisGeNET database^61^. By doing so, we intended to identify in an unbiased manner, putative disease associations with CMV infection, thereby generating testable hypotheses for future studies. To conduct the analysis, we used the disease_enrichment function with the “ALL” database of DisGeNET. The top 50 enriched diseases whose enrichment was driven by at least 25 genes were plotted.

Any choice of a fold change threshold (or not) in differential expression analyses is arbitrary and needs to be justified based on the goals of the study. A common justification for explicitly testing for genes with a large fold change is a belief that this will select for biologically relevant genes. However, the magnitude of fold change does not necessarily correlate with the biological importance of a gene being differentially expressed or not. Biologically important changes can occur as a result of even very small changes in RNA expression^62,63^, and can be detected with as few as three biological replicates per experimental condition^62^. Given that we aimed to conduct differential expression analyses to generate testable hypotheses for disease association regardless of the magnitude of gene expression change, differential expression analyses were performed without an explicit fold change cutoff incorporated into the model.

All R code used to conduct these hypergeometric and enrichment analyses are available from https://github.com/charlesfoster/CMV_Cerebral_Organoid_Paper.git.

### Reporting summary

Further information on research design is available in the Nature Portfolio Reporting Summary linked to this article.

### Supplementary information

Supplementary Information

Description of Additional Supplementary Files

Supplementary Data 1

Supplementary Data 2

Supplementary Data 3

Supplementary Data 4

Supplementary Data 5

Supplementary Data 6

Reporting Summary

## Data Availability

The RNA-Seq sequencing data has been submitted to the NCBI Gene Expression Omnibus (GEO) (https://www.ncbi.nlm.nih.gov/geo/) and under accession number GSE217832.

## Code availability

All code for differential expression and enrichment analyses (including hypergeometric tests) are available from https://github.com/s1corley/CMV. All R code used to conduct these hypergeometric and enrichment analyses are available from https://github.com/charlesfoster/CMV_Cerebral_Organoid_Paper.git.
