# Supplementary material for: Human cytomegalovirus (CMV) dysregulates neurodevelopmental pathways in cerebral organoids
Source: Commun Biol. 2024 Mar 19;7:340. doi: 10.1038/s42003-024-05923-1 (PMC10951402; doi:10.1038/s42003-024-05923-1)
Supplement: Supplementary file 1 — Supplementary Information [file 42003_2024_5923_MOESM1_ESM.pdf]

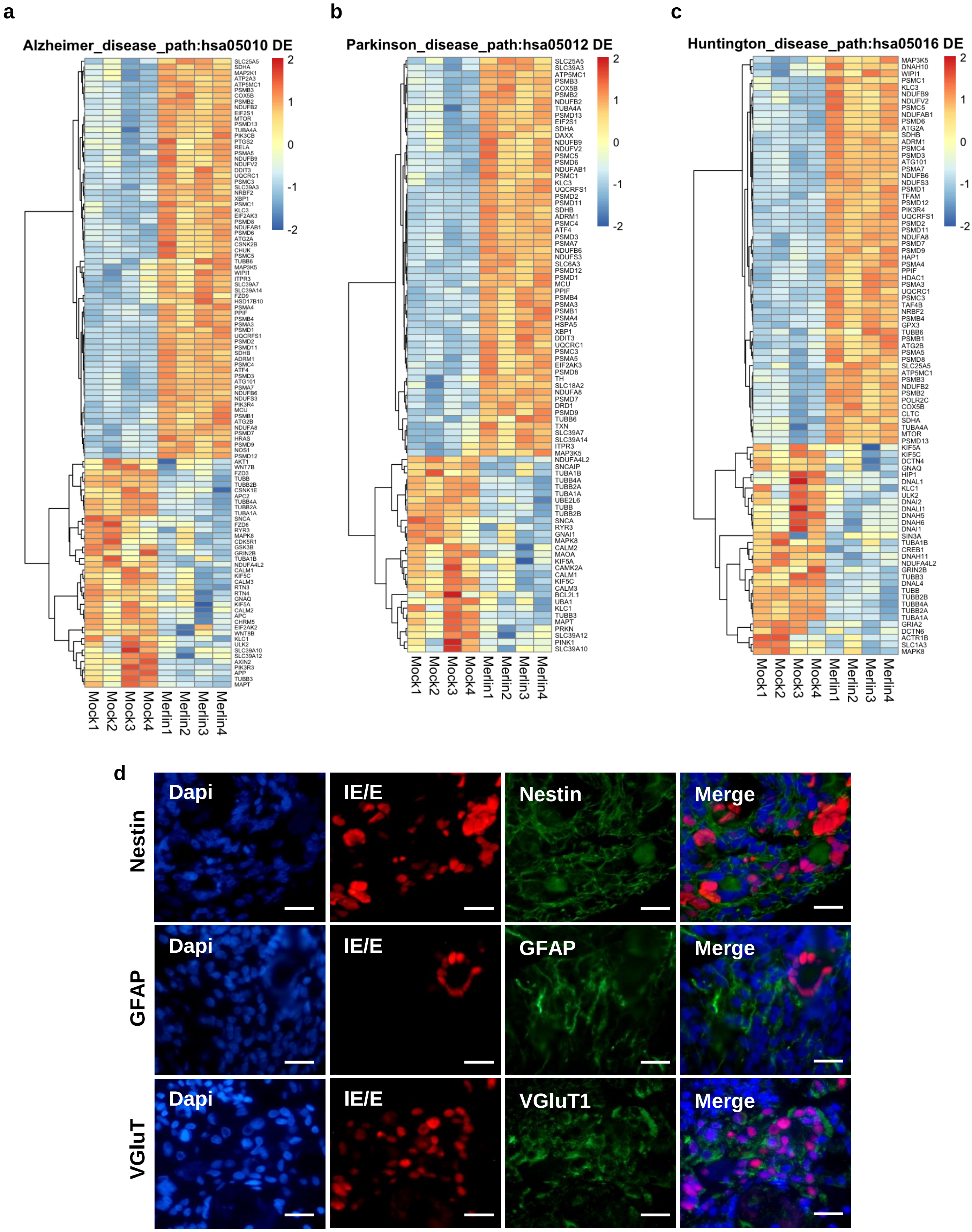

Supplementary Figure 1. Key neurodegenerative disease pathways are significantly over-repr esented in significantly upregulated DEGs of CMV-infected cerebral organoids relative to mock. Heatmaps of key KEGG pathways involved in a) Alzheimer disease (hsa05010), b) Parkinson disease (hsa05012), and c) Huntington disease (hsa05016). d) Immunofluorescence of cerebral organoids at 14dpi, co-staining for IE/E and Nestin, GFAP, and VGluT1.
