# Supplementary material for: Human cytomegalovirus (CMV) dysregulates neurodevelopmental pathways in cerebral organoids
Source: Commun Biol. 2024 Mar 19;7:340. doi: 10.1038/s42003-024-05923-1 (PMC10951402; doi:10.1038/s42003-024-05923-1)
Supplement: Supplementary file 2 — Description of Additional Supplementary Files [file 42003_2024_5923_MOESM2_ESM.pdf]

Description of Additional Supplementary Files

File name: Supplementary Data 1
Description: Differentially expressed genes in CMV-infected cerebral organoids relative to mock.

File name: Supplementary Data 2
Description: Significantly over-represented GO terms in DEGs in CMV-infected cerebral organoids relative to mock.

File name: Supplementary Data 3
Description: KEGG pathways over-represented in CMV-infected organoids relative to mock.

File name: Supplementary Data 4
Description: Differentially expressed genes in CMV-infected cerebral organoids relative to mock in DYRK and SHH pathways.

File name: Supplementary Data 5
Description: Overlapping differentially expressed genes with SFARI database.

File name: Supplementary Data 6
Description: Disease enrichment differentially expressed gene dataset using the DisGeNET database.
