# Supplementary material for: Human cytomegalovirus (CMV) dysregulates neurodevelopmental pathways in cerebral organoids
Source: Commun Biol. 2024 Mar 19;7:340. doi: 10.1038/s42003-024-05923-1 (PMC10951402; doi:10.1038/s42003-024-05923-1)
Supplement: Supplementary file 9 — Reporting Summary [file 42003_2024_5923_MOESM9_ESM.pdf]

nature portfolio

Corresponding author(s): Ece Egilmezer

Last updated by author(s): Sep 14, 2023

Reporting Summary

Nature Portfolio wishes to improve the reproducibility of the work that we publish. This form provides structure for consistency and transparency in reporting. For further information on Nature Portfolio policies, see our Editorial Policies and the Editorial Policy Checklist.

Statistics

For all statistical analyses, confirm that the following items are present in the figure legend, table legend, main text, or Methods section.

n/a	Confirmed	
☐	☒	The exact sample size (*n*) for each experimental group/condition, given as a discrete number and unit of measurement
☐	☒	A statement on whether measurements were taken from distinct samples or whether the same sample was measured repeatedly
☐	☒	The statistical test(s) used AND whether they are one- or two-sided<br>*Only common tests should be described solely by name; describe more complex techniques in the Methods section.*
☐	☒	A description of all covariates tested
☐	☒	A description of any assumptions or corrections, such as tests of normality and adjustment for multiple comparisons
☒	☐	A full description of the statistical parameters including central tendency (e.g. means) or other basic estimates (e.g. regression coefficient) AND variation (e.g. standard deviation) or associated estimates of uncertainty (e.g. confidence intervals)
☐	☒	For null hypothesis testing, the test statistic (e.g. *F*, *t*, *r*) with confidence intervals, effect sizes, degrees of freedom and *P* value noted<br>*Give P values as exact values whenever suitable.*
☒	☐	For Bayesian analysis, information on the choice of priors and Markov chain Monte Carlo settings
☒	☐	For hierarchical and complex designs, identification of the appropriate level for tests and full reporting of outcomes
☒	☐	Estimates of effect sizes (e.g. Cohen's *d*, Pearson's *r*), indicating how they were calculated

Our web collection on statistics for biologists contains articles on many of the points above.

Software and code

Policy information about availability of computer code

Data collection	Data collection is detailed in the methods of the manuscript.
Data analysis	Programs to analyse the data in this study are as follows: quality control of reads: FastQC v0.11.8; read mapping: Subread v1.6.3; general scripting/programming language: R v4.1.2; R packages used for data exploration, analysis and visualisation: edgeR v3.38.4, disgenet2r v0.99.2, data.table v1.14.2, dplyr v1.0.9, org.Hs.eg.db v3.14.0, clusterProfiler v3.4.0, ggplot2 v4.2.2, limma v3_52.3, statmod v1.5.0, gplots v3.1.3, biomaRt v2.50.3, RColorBrewer v1.1.3, tidyverse v1.3.2, pheatmap v1.0.12. All R code used to conduct these analyses is available from https://github.com/charlesfoster/CMV_Cerebral_Organoid_Paper

For manuscripts utilizing custom algorithms or software that are central to the research but not yet described in published literature, software must be made available to editors and reviewers. We strongly encourage code deposition in a community repository (e.g. GitHub). See the Nature Portfolio guidelines for submitting code & software for further information.

Data

Policy information about availability of data

All manuscripts must include a data availability statement. This statement should provide the following information, where applicable:

- Accession codes, unique identifiers, or web links for publicly available datasets
- A description of any restrictions on data availability
- For clinical datasets or third party data, please ensure that the statement adheres to our policy

The RNA-Seq sequencing data has been submitted to the NCBI Gene Expression Omnibus (GEO) (https://www.ncbi.nlm.nih.gov/geo/) and under accession number GSE217832. All code for differential expression and enrichment analyses (including hypergeometric tests) are available from https://github.com/charlesfoster/CMV_Cerebral_Organoid_Paper

Research involving human participants, their data, or biological material

Policy information about studies with human participants or human data. See also policy information about sex, gender (identity/presentation), and sexual orientation and race, ethnicity and racism.

Reporting on sex and gender	n/a
Reporting on race, ethnicity, or other socially relevant groupings	n/a
Population characteristics	n/a
Recruitment	n/a
Ethics oversight	n/a

Note that full information on the approval of the study protocol must also be provided in the manuscript.

Field-specific reporting

Please select the one below that is the best fit for your research. If you are not sure, read the appropriate sections before making your selection.

☒ Life sciences ☐ Behavioural & social sciences ☐ Ecological, evolutionary & environmental sciences

For a reference copy of the document with all sections, see nature.com/documents/nr-reporting-summary-flat.pdf

Life sciences study design

All studies must disclose on these points even when the disclosure is negative.

Sample size	A sample size of 4 replicates for each organoid condition (i.e. mock and infected) was deemed sufficient as generally, the number of replicates used for these experiments is 3 for statistical analyses.
Data exclusions	No data were excluded.
Replication	4 replicates of each sample were performed for RNA-bulk sequencing.
Randomization	Allocation of samples was not random as this is not relevent to the study.
Blinding	Blinding was not relevant to the study as these are infection experiments that require the worker's knowledge of mock vs. infected tissue.

Behavioural & social sciences study design

All studies must disclose on these points even when the disclosure is negative.

Study description	n/a
Research sample	n/a
Sampling strategy	n/a

Data collection	n/a
Timing	n/a
Data exclusions	n/a
Non-participation	n/a
Randomization	n/a

Ecological, evolutionary & environmental sciences study design

All studies must disclose on these points even when the disclosure is negative.

Study description	n/a
Research sample	n/a
Sampling strategy	n/a
Data collection	n/a
Timing and spatial scale	n/a
Data exclusions	n/a
Reproducibility	n/a
Randomization	n/a
Blinding	n/a

Did the study involve field work? ☐ Yes ☒ No

Field work, collection and transport

Field conditions	n/a
Location	n/a
Access & import/export	n/a
Disturbance	n/a

Reporting for specific materials, systems and methods

We require information from authors about some types of materials, experimental systems and methods used in many studies. Here, indicate whether each material, system or method listed is relevant to your study. If you are not sure if a list item applies to your research, read the appropriate section before selecting a response.

Materials & experimental systems

n/a	Involved in the study	
☐	☒	Antibodies
☐	☒	Eukaryotic cell lines
☒	☐	Palaeontology and archaeology
☒	☐	Animals and other organisms
☒	☐	Clinical data
☒	☐	Dual use research of concern
☒	☐	Plants

Methods

n/a	Involved in the study	
☒	☐	ChIP-seq
☒	☐	Flow cytometry
☒	☐	MRI-based neuroimaging

Antibodies

Antibodies used	IE/E; clones DDG9 and CCH2; Dako, mAb-pp28 (Abcam), rabbit mAb-Nestin (Abcam), mouse mAb-beta III Tubulin (Abcam), rabbit pAb-GFAP (Abcam), rabbit pAb-FOXG1 (Abcam), rabbit pAb-TBR1 (Abcam), rabbit mAb-VGluT1 (Abcam), pAb-DYRK1A (Abcam), rabbit mAb-DYRK1B (Abcam), rabbit mAb-Sonic Hedgehog (Abcam), rabbit mAb-ULK3 (Abcam), rabbit mAb-Rb (Abcam), rabbit pAb-Gli2 (Abcam), Alexa Fluor 488 goat anti-mouse and 594 goat donkey anti-rabbit (Invitrogen)
Validation	IE/E; clones DDG9 and CCH2: Hamilton ST, Scott G, Naing Z, Iwasenko J, Hall B, Graf N, Arbuckle S, Craig ME, Rawlinson WD. Human cytomegalovirus-induces cytokine changes in the placenta with implications for adverse pregnancy outcomes. PLoS One. 2012;7(12):e52899. doi: 10.1371/journal.pone.0052899. Epub 2012 Dec 31. PMID: 23300810; PMCID: PMC3534118. mAb-pp28 (Abcam): "Abcam is leading the way in addressing this with our range of recombinant monoclonal antibodies and knockout edited cell lines for gold-standard validation. Specificity: Specificity: Cytomegalovirus pp28 tegument protein. Slight cross reactivity with HSV1." rabbit mAb-Nestin (Abcam): "Produced recombinantly (animal-free) for high batch-to-batch consistency and long term security of supply." mouse mAb-beta III Tubulin (Abcam): "Abcam is leading the way in addressing this with our range of recombinant monoclonal antibodies and knockout edited cell lines for gold-standard validation." rabbit pAb-GFAP (Abcam): "Produced recombinantly (animal-free) for high batch-to-batch consistency and long term security of supply." rabbit pAb-FOXG1 (Abcam): "Abcam is leading the way in addressing this with our range of recombinant monoclonal antibodies and knockout edited cell lines for gold-standard validation." rabbit pAb-TBR1 (Abcam): "Abcam is leading the way in addressing this with our range of recombinant monoclonal antibodies and knockout edited cell lines for gold-standard validation." rabbit mAb-VGluT1 (Abcam): "Produced recombinantly (animal-free) for high batch-to-batch consistency and long term security of supply".<br>pAb-DYRK1A (Abcam): "Abcam is leading the way in addressing this with our range of recombinant monoclonal antibodies and knockout edited cell lines for gold-standard validation. Specificity: ab180910 will not cross-react with other DYRK family members." rabbit mAb-DYRK1B (Abcam): "Abcam is leading the way in addressing this with our range of recombinant monoclonal antibodies and knockout edited cell lines for gold-standard validation." rabbit mAb-Sonic Hedgehog (Abcam): "Produced recombinantly (animal-free) for high batch-to-batch consistency and long term security of supply. Specificity: for both full length (51kDa) and c-product subunit (27kDa) of human Sonic Hedgehog protein." rabbit mAb-ULK3 (Abcam): "Produced recombinantly (animal-free) for high batch-to-batch consistency and long term security of supply." rabbit mAb-Rb (Abcam): "Produced recombinantly (animal-free) for high batch-to-batch consistency and long term security of supply." rabbit pAb-Gli2 (Abcam): Discontinued product. Positive feedback of SuFu negating protein 1 on Hedgehog signaling promotes colorectal tumor growth. In Cell Death & Disease on 19 February 2021 by Yan, Z., Cheng, M., et al.

Eukaryotic cell lines

Policy information about cell lines and Sex and Gender in Research

Cell line source(s)	IE/E; clones DDG9 and CCH2: Hamilton ST, Scott G, Naing Z, Iwasenko J, Hall B, Graf N, Arbuckle S, Craig ME, Rawlinson WD. Human cytomegalovirus-induces cytokine changes in the placenta with implications for adverse pregnancy outcomes. PLoS One. 2012;7(12):e52899. doi: 10.1371/journal.pone.0052899. Epub 2012 Dec 31. PMID: 23300810; PMCID: PMC3534118.<br>mAb-pp28 (Abcam): "Abcam is leading the way in addressing this with our range of recombinant monoclonal antibodies and knockout edited cell lines for gold-standard validation. Specificity: Specificity: Cytomegalovirus pp28 tegument protein. Slight cross reactivity with HSV1."<br>rabbit mAb-Nestin (Abcam): "Produced recombinantly (animal-free) for high batch-to-batch consistency and long term security of supply."<br>mouse mAb-beta III Tubulin (Abcam): "Abcam is leading the way in addressing this with our range of recombinant monoclonal antibodies and knockout edited cell lines for gold-standard validation."<br>rabbit pAb-GFAP (Abcam): "Abcam is leading the way in addressing this with our range of recombinant monoclonal antibodies and knockout edited cell lines for gold-standard validation. Specificity: recognizes mammalian GFAP on western blots and immunocytochemically. Detects a band of 55kDa corresponding to GFAP and also a GFAP derived 48kDa band."<br>rabbit pAb-FOXG1 (Abcam): "Abcam is leading the way in addressing this with our range of recombinant monoclonal antibodies and knockout edited cell lines for gold-standard validation."<br>rabbit pAb-TBR1 (Abcam): "Abcam is leading the way in addressing this with our range of recombinant monoclonal antibodies and knockout edited cell lines for gold-standard validation."<br>pAb-DYRK1A (Abcam): "Abcam is leading the way in addressing this with our range of recombinant monoclonal antibodies and knockout edited cell lines for gold-standard validation. Specificity: ab180910 will not cross-react with other DYRK family members."<br>rabbit mAb-DYRK1B (Abcam): "Abcam is leading the way in addressing this with our range of recombinant monoclonal antibodies and knockout edited cell lines for gold-standard validation."<br>rabbit mAb-Sonic Hedgehog (Abcam): "Produced recombinantly (animal-free) for high batch-to-batch consistency and long term security of supply. Specificity: for both full length (51kDa) and c-product subunit (27kDa) of human Sonic Hedgehog protein."<br>rabbit mAb-ULK3 (Abcam): "Produced recombinantly (animal-free) for high batch-to-batch consistency and long term security of supply."<br>rabbit mAb-Rb (Abcam): "Produced recombinantly (animal-free) for high batch-to-batch consistency and long term security of supply."<br>rabbit pAb-Gli2 (Abcam): Discontinued product. Positive feedback of SuFu negating protein 1 on Hedgehog signaling promotes colorectal tumor growth. In Cell Death & Disease on 19 February 2021 by Yan, Z., Cheng, M., et al.<br>IE/E; clones DDG9 and CCH2: Hamilton ST, Scott G, Naing Z, Iwasenko J, Hall B, Graf N, Arbuckle S, Craig ME, Rawlinson WD. Human cytomegalovirus-induces cytokine changes in the placenta with implications for adverse pregnancy outcomes. PLoS One. 2012;7(12):e52899. doi: 10.1371/journal.pone.0052899. Epub 2012 Dec 31. PMID: 23300810; PMCID: PMC3534118.<br>mAb-pp28 (Abcam): "Abcam is leading the way in addressing this with our range of recombinant monoclonal antibodies and knockout edited cell lines for gold-standard validation. Specificity: Specificity: Cytomegalovirus pp28 tegument protein. Slight cross reactivity with HSV1."<br>rabbit mAb-Nestin (Abcam): "Produced recombinantly (animal-free) for high batch-to-batch consistency and long term

security of supply."
mouse mAb-beta III Tubulin (Abcam): "Abcam is leading the way in addressing this with our range of recombinant monoclonal antibodies and knockout edited cell lines for gold-standard validation."
rabbit pAb-GFAP (Abcam): "Abcam is leading the way in addressing this with our range of recombinant monoclonal antibodies and knockout edited cell lines for gold-standard validation. Specificity: recognizes mammalian GFAP on western blots and immunocytochemically. Detects a band of 55kDa corresponding to GFAP and also a GFAP derived 48kDa band."
rabbit pAb-FOXG1 (Abcam): "Abcam is leading the way in addressing this with our range of recombinant monoclonal antibodies and knockout edited cell lines for gold-standard validation."
rabbit pAb-TBR1 (Abcam): "Abcam is leading the way in addressing this with our range of recombinant monoclonal antibodies and knockout edited cell lines for gold-standard validation."
pAb-DYRK1A (Abcam): "Abcam is leading the way in addressing this with our range of recombinant monoclonal antibodies and knockout edited cell lines for gold-standard validation. Specificity: ab180910 will not cross-react with other DYRK family members."
rabbit mAb-DYRK1B (Abcam): "Abcam is leading the way in addressing this with our range of recombinant monoclonal antibodies and knockout edited cell lines for gold-standard validation."
rabbit mAb-Sonic Hedgehog (Abcam): "Produced recombinantly (animal-free) for high batch-to-batch consistency and long term security of supply. Specificity: for both full length (51kDa) and c-product subunit (27kDa) of human Sonic Hedgehog protein."
rabbit mAb-ULK3 (Abcam): "Produced recombinantly (animal-free) for high batch-to-batch consistency and long term security of supply."
rabbit mAb-Rb (Abcam): "Produced recombinantly (animal-free) for high batch-to-batch consistency and long term security of supply."
rabbit pAb-Gli2 (Abcam): Discontinued product. Positive feedback of SuFu negating protein 1 on Hedgehog signaling promotes colorectal tumor growth. In Cell Death & Disease on 19 February 2021 by Yan, Z., Cheng, M., et al.
IE/E; clones DDG9 and CCH2: Hamilton ST, et al. . Human cytomegalovirus-induces cytokine changes in the placenta with implications for adverse pregnancy outcomes. PLoS One.
mAb-pp28 (Abcam): "Abcam is leading the way in addressing this with our range of recombinant monoclonal antibodies and knockout edited cell lines for gold-standard validation. Specificity: Specificity: Cytomegalovirus pp28 tegument protein. Slight cross reactivity with HSV1."
rabbit mAb-Nestin (Abcam): "Produced recombinantly (animal-free) for high batch-to-batch consistency and long term security of supply."
mouse mAb-beta III Tubulin (Abcam): "Abcam is leading the way in addressing this with our range of recombinant monoclonal antibodies and knockout edited cell lines for gold-standard validation."
rabbit pAb-GFAP (Abcam): "Abcam is leading the way in addressing this with our range of recombinant monoclonal antibodies and knockout edited cell lines for gold-standard validation. Specificity: recognizes mammalian GFAP on western blots and immunocytochemically. Detects a band of 55kDa corresponding to GFAP and also a GFAP derived 48kDa band."
rabbit pAb-FOXG1 (Abcam): "Abcam is leading the way in addressing this with our range of recombinant monoclonal antibodies and knockout edited cell lines for gold-standard validation."
rabbit pAb-TBR1 (Abcam): "Abcam is leading the way in addressing this with our range of recombinant monoclonal antibodies and knockout edited cell lines for gold-standard validation."
pAb-DYRK1A (Abcam): "Abcam is leading the way in addressing this with our range of recombinant monoclonal antibodies and knockout edited cell lines for gold-standard validation. Specificity: ab180910 will not cross-react with other DYRK family members."
rabbit mAb-DYRK1B (Abcam): "Abcam is leading the way in addressing this with our range of recombinant monoclonal antibodies and knockout edited cell lines for gold-standard validation."
rabbit mAb-Sonic Hedgehog (Abcam): "Produced recombinantly (animal-free) for high batch-to-batch consistency and long term security of supply. Specificity: for both full length (51kDa) and c-product subunit (27kDa) of human Sonic Hedgehog protein."
Human Episomal iPSC Line

Authentication

None of the cell lines were authenticated. This cell line was purchased directly from Life Technologies

Mycoplasma contamination

Tested for Mycoplasma regularly

Commonly misidentified lines
(See ICLAC register)

None.

Palaeontology and Archaeology

Specimen provenance

n/a

Specimen deposition

n/a

Dating methods

n/a

☐ Tick this box to confirm that the raw and calibrated dates are available in the paper or in Supplementary Information.

Ethics oversight

n/a

Note that full information on the approval of the study protocol must also be provided in the manuscript.

Animals and other research organisms

Policy information about studies involving animals; ARRIVE guidelines recommended for reporting animal research, and Sex and Gender in Research

Laboratory animals	n/a
Wild animals	n/a
Reporting on sex	n/a
Field-collected samples	n/a
Ethics oversight	n/a

Note that full information on the approval of the study protocol must also be provided in the manuscript.

Clinical data

Policy information about clinical studies

All manuscripts should comply with the ICMJE guidelines for publication of clinical research and a completed CONSORT checklist must be included with all submissions.

Clinical trial registration	n/a
Study protocol	n/a
Data collection	n/a
Outcomes	n/a

Dual use research of concern

Policy information about dual use research of concern

Hazards

Could the accidental, deliberate or reckless misuse of agents or technologies generated in the work, or the application of information presented in the manuscript, pose a threat to:

No	Yes	
☒	☐	Public health
☒	☐	National security
☒	☐	Crops and/or livestock
☒	☐	Ecosystems
☒	☐	Any other significant area

Experiments of concern

Does the work involve any of these experiments of concern:

No	Yes	
☒	☐	Demonstrate how to render a vaccine ineffective
☒	☐	Confer resistance to therapeutically useful antibiotics or antiviral agents
☒	☐	Enhance the virulence of a pathogen or render a nonpathogen virulent
☒	☐	Increase transmissibility of a pathogen
☒	☐	Alter the host range of a pathogen
☒	☐	Enable evasion of diagnostic/detection modalities
☐	☒	Enable the weaponization of a biological agent or toxin
☒	☐	Any other potentially harmful combination of experiments and agents

Precautions and benefits

Biosecurity precautions | *Describe the precautions that were taken during the design and conduct of this research, or will be required in the communication and application of the research, to minimise biosecurity risks. These may include bio-containment facilities, changes to the study design/ methodology or redaction of details from the manuscript.*

Biosecurity oversight | *Describe any evaluations and oversight of biosecurity risks of this work that you have received from people or organizations outside of your immediate team.*

Benefits | *Describe the benefits that application or use of this work could bring, including benefits that may mitigate risks to public health, national security, or the health of crops, livestock or the environment.*

Communication benefits | *Describe whether the benefits of communicating this information outweigh the risks, and if so, how.*

Plants

Seed stocks | n/a

Novel plant genotypes | n/a

Authentication | n/a

ChIP-seq

Data deposition

☐ Confirm that both raw and final processed data have been deposited in a public database such as GEO.

☐ Confirm that you have deposited or provided access to graph files (e.g. BED files) for the called peaks.

Data access links
May remain private before publication. | n/a

Files in database submission | n/a

Genome browser session (e.g. UCSC) | n/a

Methodology

Replicates | n/a

Sequencing depth | n/a

Antibodies | n/a

Peak calling parameters | n/a

Data quality | n/a

Software | n/a

Flow Cytometry

Plots

Confirm that:

☐ The axis labels state the marker and fluorochrome used (e.g. CD4-FITC).

☐ The axis scales are clearly visible. Include numbers along axes only for bottom left plot of group (a 'group' is an analysis of identical markers).

☐ All plots are contour plots with outliers or pseudocolor plots.

☐ A numerical value for number of cells or percentage (with statistics) is provided.

Methodology

Sample preparation | n/a

Instrument | n/a

Software	n/a
Cell population abundance	n/a
Gating strategy	n/a

☐ Tick this box to confirm that a figure exemplifying the gating strategy is provided in the Supplementary Information.

Magnetic resonance imaging

Experimental design

Design type	n/a
Design specifications	n/a
Behavioral performance measures	n/a

Acquisition

Imaging type(s)	n/a
Field strength	n/a
Sequence & imaging parameters	n/a
Area of acquisition	n/a

Diffusion MRI ☐ Used ☒ Not used

Preprocessing

Preprocessing software	n/a
Normalization	n/a
Normalization template	n/a
Noise and artifact removal	n/a
Volume censoring	n/a

Statistical modeling & inference

Model type and settings	n/a
Effect(s) tested	n/a

Specify type of analysis: ☐ Whole brain ☐ ROI-based ☐ Both

Statistic type for inference (See Eklund et al. 2016)	n/a
Correction	n/a

Models & analysis

n/a	Involved in the study
☒	☐ Functional and/or effective connectivity
☒	☐ Graph analysis
☒	☐ Multivariate modeling or predictive analysis

Functional and/or effective connectivity	n/a
Graph analysis	n/a

Multivariate modeling and predictive analysis	n/a
